# Application of Doehlert design combined with chemometrics tools: Example of the optimization of the elution of neurotransmitters and metabolites by HPLC

**DOI:** 10.1016/j.heliyon.2025.e42690

**Published:** 2025-02-14

**Authors:** Camille Rosier, Bruno P. Guiard, Guillaume Gotti

**Affiliations:** a Centre de recherches sur la Cognition Animale (CRCA), CNRS UMR 5169, 31062, Toulouse, France; b Centre de Biologie Intégrative (CBI), Université de Toulouse III, Faculté Sciences Ingénierie (FSI), 31062, Toulouse, France

**Keywords:** Doehlert experimental design, Chemometrics, HPLC, Electrochemical detection, Neurotransmitters, Pearson correlation

## Abstract

Experimental designs are essential mathematical tools in fields like agronomy, chemistry, and analytical chemistry for optimizing processes and minimizing variations. Doehlert designs, in particular, are valued for their efficiency in exploring experimental space with minimal experiments, providing detailed insights into complex processes. In analytical chemistry, these designs are extensively used for tasks such as extraction, purification, and method optimization, allowing systematic variation of factors to enhance accuracy and efficiency. To further optimize the method, a combination of experimental design and chemometric tools is necessary to understand the chromatographic behavior of the compounds of interest. This study uses Doehlert experimental design combined with chemometric tools to optimize the elution and separation of neurotransmitters and their metabolites using HPLC-ECD. Pearson correlation and Partial Least Squares Discriminant Analysis (PLS-DA) reveal significant relationships between chromatographic parameters and experimental conditions. Notably, the pH of the mobile phase significantly impacts column efficiency and elution time, while the polarity index and pressure influence peak asymmetry. Optimized conditions include a mobile phase of ACN/MeOH/H_2_O (11.25/3.25/85.5; v/v/v) with a pH near 1.65, achieving optimal elution times around 20 min, column efficiency with a mean number of theoretical plates close to 8000, and peak asymmetry of approximately 1.23. The limits of detection and quantification of interest compounds are close to 10^−11^ and 10^−10^ mol L^−1^ respectively. This new combined approach allows for effective, rapid, and resolved elution of compounds, reducing resource consumption and time. Moreover, the combination of parameters has been taken into account with chemometrics, allowing a highly effective enhancement of compound elution. The optimized method achieves low detection and quantification limits in the nanomolar range, making it suitable for precise neurotransmitter analysis in complex biological samples.

## 1 Introduction

Experimental designs are mathematical tools widely used in various fields such as agronomy [1], chemistry [2] and others [3,4]. These mathematic tools like those of Doehlert, Taguchi [5], and Box-Behnken [6] offer robust methodological frameworks to minimize variations, and maximize performance, thus playing an essential role in research and development. Although various mathematical models rely on Hadamard matrices, the Doehlert experimental design requires fewer experiments compared to other mathematical designs such as the Box-Behnken or Taguchi methods.

Doehlert experimental designs are widely used in scientific research due to their efficiency in exploring experimental space with few experiments, thus enabling comprehensive insights into complex processes. This mathematical design was described by Doehlert in 1970 [7]. The flexibility is relatively high to simultaneously explore multiple variables and effectively optimize experimental conditions. Despite these advantages, difficulties may arise in handling nonlinear relationships or interactions among factors. That could require conducting additional experiments or exploring alternative strategies. Additionally, it is crucial to carefully examine assumptions and biases when relying on mathematical models for optimization. Therefore, although these methods offer benefits for systematic exploration and optimization, it is necessary to evaluate their relevance and limitations in specific contexts [8].

In analytical chemistry, Doehlert designs are extensively utilized, particularly for tasks such as extraction, purification, method development, and optimization [9,10]. These designs allow researchers to systematically vary factors such as pH, temperature, and reactant concentrations, effectively investigating their effects on sensitivity, selectivity, and resolution [10]. This systematic approach aids in identifying optimal conditions, thereby improving accuracy and efficiency. Additionally, Doehlert designs assess interactions between variables, revealing complex relationships within analytical processes. Their ability to conduct experiments with a minimal number of tests, as indicated in Eq. (1), reduces resource consumption and time, making them suitable for laboratories with limited resources [11].

(1) $$n=k^{2}+k+1$$

where n is the number of experiments and k the number of parameters.

Quantifying levels of neurotransmitters, notably by HPLC-ECD [12], is a crucial aspect of research in neuroscience, impacting the understanding of various physiological and cognitive processes [[13], [14], [15]]. To enhance the precision and efficiency of neurotransmitter analysis, experimental designs such as Doehlert can be employed, especially during the analysis of samples from intracerebral microdialysis [16], where the samples can be complex and contain extracellular concentrations of neurotransmitters and metabolites on the order of femtomoles [17].

After data collection, chemometric analysis must be conducted to determine optimum elution parameters. For instance, Pearson correlation or Partial Least Squares Discriminant Analysis (PLS-DA) [18] can be used to explore relationships between measured neurotransmitter concentrations and experimental variables. Significant associations can be identified, guiding the refinement of experimental protocols.

In our study, we propose to use a Doehlert experimental design followed by several chemometric tools. This innovative combination of mathematical tools will optimize the analytical method for eluting neurotransmitters and their metabolites, as well as their separation and quantification using HPLC-ECD.

## 2 Materials & methods

### 2.1 Chemicals & solutions

Main text HPLC-grade methanol (MeOH) and acetonitrile (ACN) were obtained from Carlo Erba (Cornaredo, IT). Sodium dodecyl sulfate (SDS) (anhydrous ACS reagent ≥99 %), orthophosphoric acid (H_3_PO_4_) (ACS reagent, ≥85 % wt. in H_2_O), sodium phosphate monobasic (NaH_2_PO_4_) (Reagent- Plus® ≥ 99 %), sodium phosphate dibasic (Na_2_HPO_4_) (ACS reagent, ≥99 %), citric acid (ACS Reagent ≥99,5 %), ethylenediaminetetraacetic acid (EDTA) (ACS reagent, ≥99,5 %) and ascorbic acid (ACS Reagent ≥99 %) were obtained from Sigma–Aldrich Merck (Darmstad, DE). All standards used were purchased from Sigma–Aldrich Merck (Darmstad, DE): serotonin (5-HT) (Analytical Standard), (−)-norepinephrine (NE) (≥98 % crystalline), dopamine (DA) (certified reference material, Trace CERT®), melatonin (MT) (≥98 % TLC), 5-hydroxyindole-3-acetic acid (5-HIAA) (≥98 % HPLC), (−)-epinephrine (E) (HPLC Grade), 3,4-dihydroxyphenylacetic acid (DOPAC) (98 %), 3,4-dihydroxyphényl-L-alanine (L-DOPA) (≥98 % TLC) and homovanillic acid (HVA) (Analytical Standard). All solutions used were prepared with ultra-pure water (18.2 MΩ cm minimum).

### 2.2 HPLC equipment

The analysis was performed on an Ultimate 3000 HPLC system (ThermoFisher Scientific, Waltham USA) equipped with an electrochemical detector. The analytical column used was an Accucore C_18_ (50 × 3 mm, 2.6 μm particle size) fitted with a C_18_ guard cartridge both purchased from ThermoFisher Scientific (Waltham USA). The flow rate of the mobile phase was fixed at 0.5 mL min^−1^ obtained with an ISO 3100 BM isocratic pump. The mobile phase consisted of 5 % ACN, 10 % MeOH and 85 % of a buffer solution which contained 20 mmol L^−1^ of H_3_PO_4_, 130 mmol L^−1^ of NaH_2_PO_4_, 4.76 mmol L^−1^ of citric acid, 2 mmol L^−1^ of SDS and 50 μmol L^−1^ of EDTA. The pH of this solution was measured and was 3.1 ± 0.1. The mobile phase was filtered through a 0.22 μm cellulose membrane. The column oven temperature was set at room temperature and the run time was 15 min. The electrochemical detector employed was an ECD 3000 equipped with a 6041 RS ultra Amperometric Analytical Cell, which consisted of an amperometric module composed of a glassy carbon electrode operating at a potential of +550 mV vs. NHE, and the internal volume of the module was 50 nL. The injection volume was 25 μL acquired with a WPS 3000 TBPL Analytical autosampler. These conditions have been used as a basis for carrying out the development of the method as previously published [19].

### 2.3 Doehlert design

The Doehlert design used in this study has 5 parameters, all of which contribute to the composition of the eluent. Table 1 gathers the extreme values of the 5 parameters, as well as the step and the center, which are used for the calculation of the applied experimental values Y (Table 2). The Doehlert coefficient X used for the calculation of Y has been reported in supporting information file 1.

**Table 1:** Extreme values of the parameters used in the Doehlert experimental design.

	ACN (%)	MeOH (%)	pH	SDS (mol L^−1^)	Citric acid (mol L^−1^)
Min	0	0	2	1.5 10^−3^	2 10^−3^
Max	15	15	5	2.5 10^−3^	8 10^−3^
Center	7.5	7.5	3.5	2.0 10^−3^	5 10^−3^
Step	7.5	7.5	1.5	0.5 10^−3^	3 10^−3^

**Table 2:** Experimental design (Y values).

	ACN (%)	MeOH (%)	pH	SDS (mol L^−1^)	Citric acid (mol L^−1^)
1	7.50	7.50	3.500	2.000 10^−3^	5.000 10^−3^
2	15.00	7.50	3.500	2.000 10^−3^	5.000 10^−3^
3	11.25	14.00	3.500	2.000 10^−3^	5.000 10^−3^
4	3.75	14.00	3.500	2.000 10^−3^	5.000 10^−3^
5	0.00	7.50	3.500	2.000 10^−3^	5.000 10^−3^
6	3.75	1.01	3.500	2.000 10^−3^	5.000 10^−3^
7	11.25	1.01	3.500	2.000 10^−3^	5.000 10^−3^
8	11.25	9.67	4.724	2.000 10^−3^	5.000 10^−3^
9	3.75	9.67	4.724	2.000 10^−3^	5.000 10^−3^
10	7.50	3.17	4.724	2.000 10^−3^	5.000 10^−3^
11	11.25	5.33	2.276	2.000 10^−3^	5.000 10^−3^
12	3.75	5.33	2.276	2.000 10^−3^	5.000 10^−3^
13	7.50	11.83	2.276	2.000 10^−3^	5.000 10^−3^
14	11.25	9.67	3.806	2.396 10^−3^	5.000 10^−3^
15	3.75	9.67	3.806	2.396 10^−3^	5.000 10^−3^
16	7.50	3.17	3.806	2.396 10^−3^	5.000 10^−3^
17	7.50	7.50	2.582	2.396 10^−3^	5.000 10^−3^
18	11.25	5.33	3.194	1.605 10^−3^	5.000 10^−3^
19	3.75	5.33	3.194	1.605 10^−3^	5.000 10^−3^
20	7.50	11.83	3.194	1.605 10^−3^	5.000 10^−3^
21	7.50	7.50	4.418	1.605 10^−3^	5.000 10^−3^
22	11.25	9.67	3.806	2.079 10^−3^	7.325 10^−3^
23	3.75	9.67	3.806	2.079 10^−3^	7.325 10^−3^
24	7.50	3.17	3.806	2.079 10^−3^	7.325 10^−3^
25	7.50	7.50	2.582	2.079 10^−3^	7.325 10^−3^
26	7.50	7.50	3.500	1.684 10^−3^	7.325 10^−3^
27	11.25	5.33	3.194	1.921 10^−3^	2.675 10^−3^
28	3.75	5.33	3.194	1.921 10^−3^	2.675 10^−3^
29	7.50	11.83	3.194	1.921 10^−3^	2.675 10^−3^
30	7.50	7.50	4.418	1.921 10^−3^	2.675 10^−3^
31	7.50	7.50	3.500	2.316 10^−3^	2.675 10^−3^

Calculation of experimental value Y of each parameter as a function of coefficient in Doehlert design according to the following Eq. (2):

(2) Y=(X×S)+C

where Y is the experimental value, X the Doehlert coefficient, S the step and C the center.

### 2.4 Chemometrics

All mathematical exploitation of the Doehlert model was conducted using the software R and R Studio. The packages utilized include mixOmics, ggplot2, and MASS. No data was excluded from the analysis of the results. Pearson correlation calculations were performed according to the following Eq. (3):

(3) $$r=\frac{\sum_{i=1}^{n}\left(x_{i}-\bar{x}\right)\left(y_{i}-\bar{y}\right)}{\sqrt{{\sum_{i=1}^{n}\left(x_{i}-\bar{x}\right)}^{2}}\sqrt{{\sum_{i=1}^{n}\left(y_{i}-\bar{y}\right)}^{2}}}$$

where r is the Pearson coefficient, n is the number of points, x_i_ is the value of x for point i, $\overset{®}{x}$ is the mean of the x values, y_i_ is the value of y for point i, and $\overset{®}{y}$ is the mean of the y values.

A Partial Least Squares Discriminant Analysis (PLS-DA) has been performed on the measured data (retention time of compounds, number of theoretical plates, peak asymmetry, and resolution) of all compounds studied. The results will provide indications on the chromatographic behavior of the 10 compounds of interest based on the chosen elution parameters.

### 2.5 Limits of detection and quantification

The determination of detection and quantification limits is based on signal-to-noise ratios equal to 3 and 10 respectively. The noise is measured for each component, and the different calibration curves for each of them are constructed according to the concentrations reported in the supporting information file 2.

## 3 Results and discussion

### 3.1 Pearson correlation

Initially, Pearson correlations were evaluated based on variable parameters used for the design of the mobile phase: percentage of MeOH in the mobile phase, percentage of ACN in the mobile phase, pH, concentration of SDS, and concentration of citric acid. The different measured parameters of chromatographic system include pressure and polarity index according to the work of Freed et al. [20], based on the following Eq. (4):

(4) $$PI=\sum_{i=0}^{n}{PI}_{i}\times {c\left(\%\right)}_{i}$$

where PI is the global polarity index of the mobile phase, PI_i_ is the polarity index of the i component and c (%)_i_ the percentage of i component in the mobile phase.

**Fig. 1:**
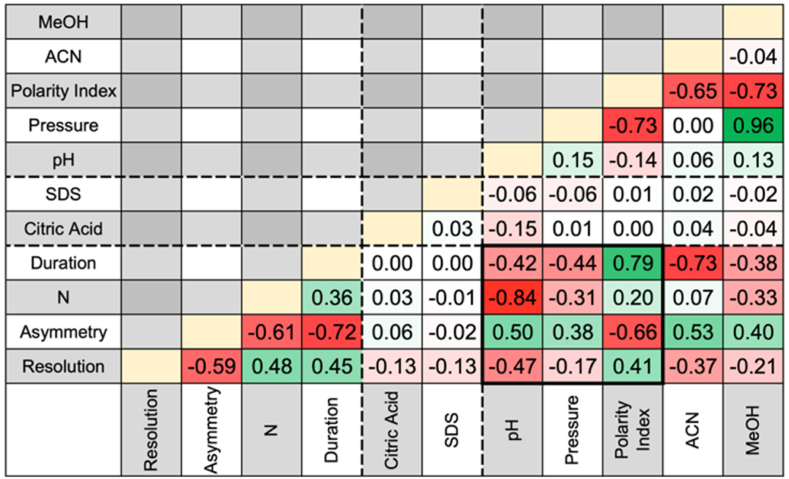
Pearson correlation r of parameters and observed characteristics. Where MeOH is the percentage of methanol in the mobile phase, ACN is the percentage of acetonitrile in mobile phase, Polarity Index is the polarity index of the mobile phase, Pressure is the system pressure, pH is the pH of the mobile phase, SDS is the concentration of SDS in mobile phase, Citric Acid is the concentration of citric acid in mobile phase, Duration is the elution duration of the run, N is the mean of number of theorical plates, Asymmetry is the mean of peaks asymmetry and Resolution is the mean of resolution.

For each compound, retention time, theoretical plate number, chromatographic peak asymmetry, and resolution between two peaks have been recorded. In Fig. 1, the correlation of Pearson between all parameters have been reported.

The polarity index variation is indeed dependent on the composition of the mobile phase and the proportions of ACN and MeOH. On the other hand, the average system pressure is strongly influenced by the proportion of MeOH (r = 0.96), which has a higher viscosity than ACN; 0.34, 0.55 and 0.89 for ACN, MeOH and H_2_O respectively [21]. For the exploitation of PLS-DA results, pressure and polarity index will be used.

The SDS and citric acid have very low influence on the elution of compounds, values in table dotted lines, correlation coefficient less than 0.15. However, it should be noted that these parameters will have an effect on the elution of compounds in biological matrices, particularly to prevent coagulation [22] or influence the elution of protonated compounds [23].

**Fig. 2:**
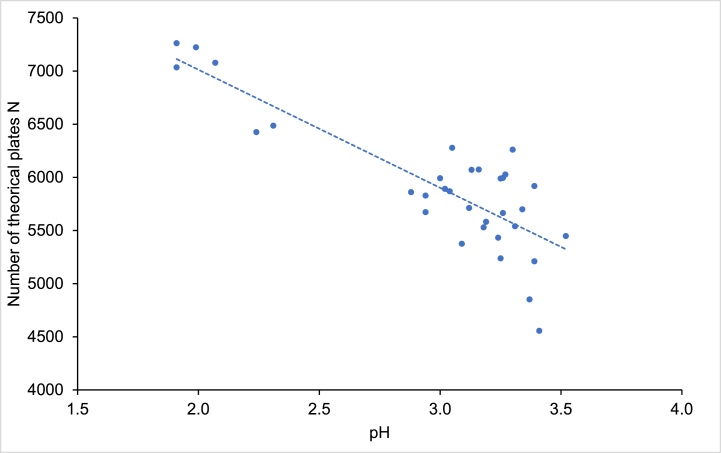
Pearson correlation between the pH of the mobile phase and the mean of the number of theorical plates measured.

The most significant correlations are those bordered by thick lines in the previous Fig. 1. The highest correlation was found between the pH of the mobile phase and the average theoretical plate number, thus the efficiency of the column (r = −0.84). This trend is depicted in Fig. 2. In order to increase the efficiency of the column, the pH of the mobile phase should ideally be acidic, preferably close to 2. A more acidic pH could compromise the stability of the column.

For the other coefficients, the elution time of the compounds is proportional to the solvent polarity index (r = 0.79), and the polarity of the mobile phase affects the peak asymmetry (r = −0.66). It will be necessary for the polarity index used to have a sufficiently high value for the separation of compounds in a relatively short time, but with the risk to increase peak asymmetry and lose sensitivity. Finally, pressure and pH increase peak asymmetry; therefore, it will be preferable to use ACN rather than MeOH to decrease the polarity index and achieve relatively low pressure compared to the use of MeOH.

### 3.2 PLS-DA analysis

A PLS-DA analysis was conducted on the elution time of compounds, the number of theoretical plates, and peak asymmetry to compare these data with the Pearson correlation values previously obtained. The purpose of this additional analysis is to identify the chromatographic behaviors of all the compounds of interest in detail.

**Fig. 3:**
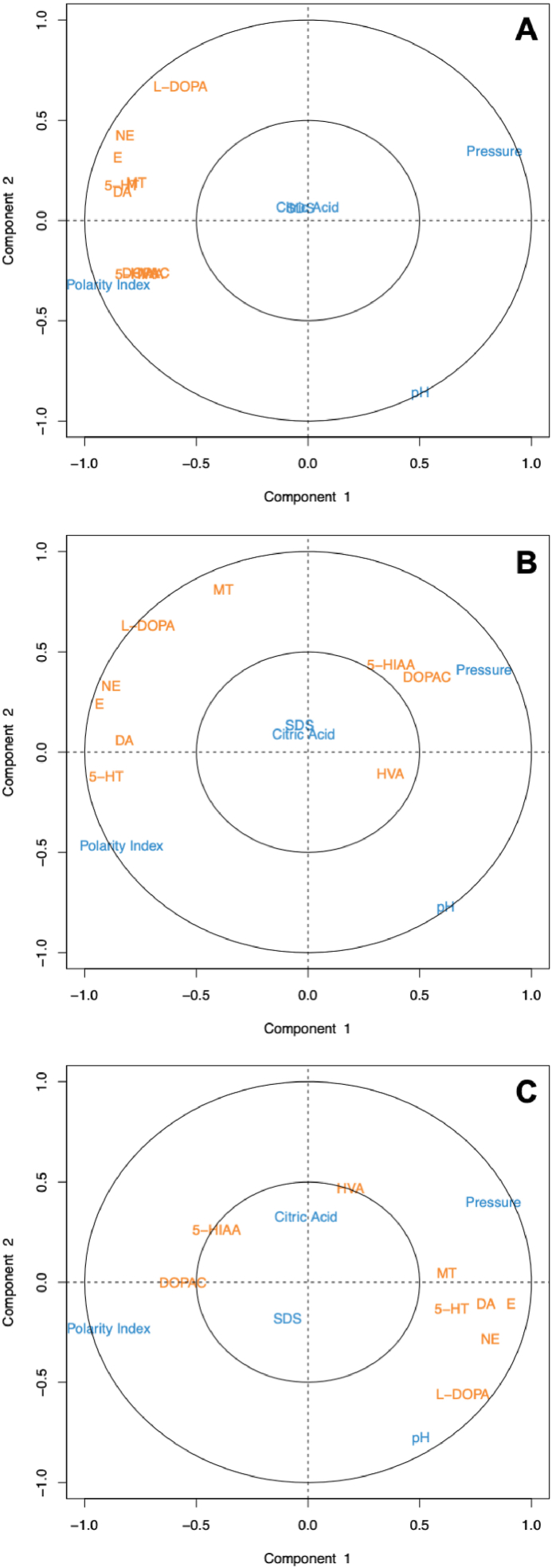
PLS-DA of the retention time of the compounds (A), the number of theorical plates (B) and the asymmetry of the peak (C).

Fig. 3 depicts the PLS-DA plots for the 9 compounds of interest (i.e. monoamines and their metabolites). Initially, retention times are analyzed in Fig. 3A. It is evident that the pH of the mobile phase has an effect on the elution of compounds, particularly on L-DOPA. For NE, E, 5-HT, MT and DA, the pH effect is similar. Conversely, the least affected compounds by pH variation are DOPA, HVA, and 5-HIAA, due to their low retention times. In Fig. 3B, the number of theoretical plates for MT, L-DOPA, NE, E, DA, and 5-HT is highly dependent on pH, unlike 5-HIAA, DOPAC, and HVA. Furthermore, a lower polarity index will lead to increased efficiency for 5-HIAA and DOPAC. Since these compounds have very low retention times, it would be beneficial to use a relatively low polarity index. Finally in Fig. 3C, the peak asymmetry of L-DOPA, NE, E, DA, 5-HT, and MT is increased when pH and/or pressure increase.

The other compounds, HVA, 5-HIAA, and DOPAC, are less sensitive to this effect. Moreover, increasing the polarity index reduces peak asymmetry.

From these various observations of Pearson correlation and PLS-DA on chromatographic behaviors, it is possible to establish optimal conditions for the elution of the studied compounds.

### 3.3 Optimized condition

The previous results, obtained through a combination of Doehlert design and chemometric tools, have highlighted the parameters to use for obtaining effective, rapid, and resolved elution of the compounds of interest:

• Preferential use of a majority of ACN over MeOH to decrease the polarity index while maintaining relatively low pressure.
• Polarity index close to 11.5.
• pH of the mobile phase close to 2: [H_3_PO_4_] = 0.15 mol L^−1^.
• SDS and citric acid about 2 10^−3^ mol L^−1^ and 5 10^−3^ mol L^−1^ respectively.

To refine the conditions, several mixtures of ACN/MeOH/H_2_O were tested. The detailed results are reported in the supplementary information file 3: for a fixed polarity index of 11.5, increasing the MeOH to ACN ratio in the mobile phase results in an increase in retention time from 15 to 35 min and a decrease in the number of theoretical plates from 8000 to 7100 for mobile phases of ACN/H_2_O (15/85 v/v) and MeOH/H_2_O (13/87 v/v), respectively. The peak asymmetry appears to be optimal when a mixture of ACN/MeOH/H_2_O is used, close to 1.23. Finally, the use of a mix containing ACN/MeOH/H_2_O (11.25/3.25/85.5; v/v/v) as the mobile phase seems to be a good compromise for achieving an elution time close to 20 min, optimal column efficiency close to 8000, and the lowest possible peak asymmetry by using a pH close to 1.65.

**Fig. 4:**
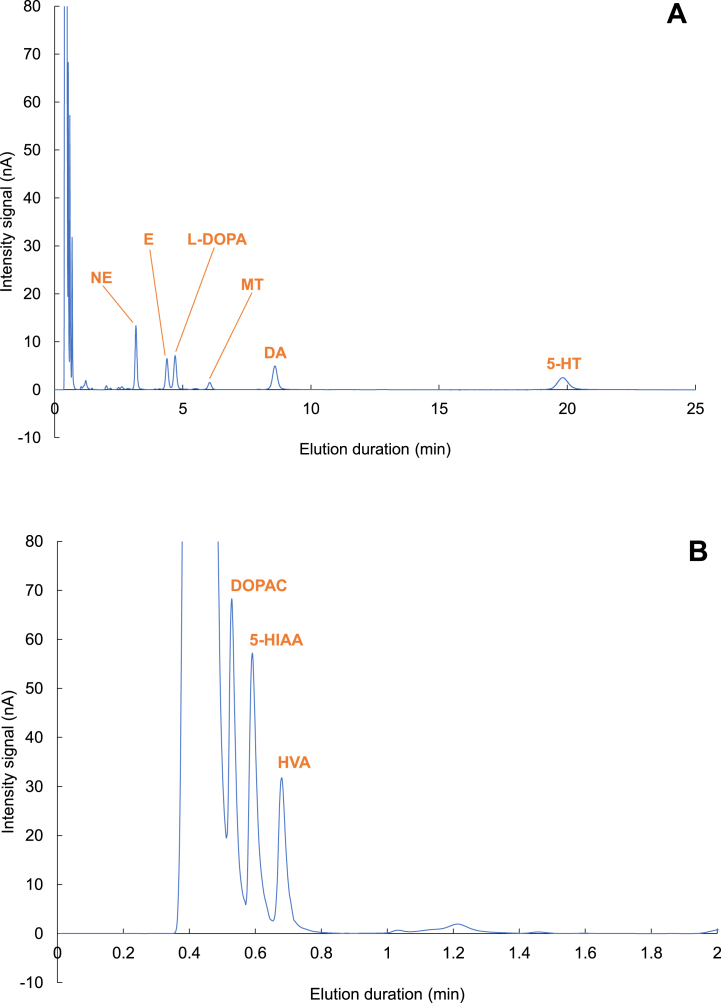
Chromatograms between 0 and 25 min (A) and 0–2 min (B) of a mixture of the 9 compounds of interest with a concentration close to 2 ng/25 μL.

Fig. 4A represents the chromatogram of a mixture of compounds of interest using the optimized conditions. With these elution conditions, the elution time is 20 min and is compatible with intracerebral microdialysis experiments for which sampling times are often close to 20 min [[24], [25], [26]]. Fig. 4B shows a zoomed-in view of the beginning of the chromatogram between 0 and 2 min. This zoom highlights the separation of 5-HIAA, DOPAC, and HVA.

### 3.4 Limits of detection and quantification

Once the elution of the compounds is optimized, the values of detection and quantification limits are calculated based on signal-to-noise ratios of 3 and 10, respectively. The various values obtained are reported in Table 3. All the calibration curves have been reported in the supporting information file 4.

**Table 3:** Values of limit of detection (LOD) and quantification (LOQ) obtained with the optimized condition.

	LOD		LOQ	
	pg/25 μL	mol L^−1^	pg/25 μL	mol L^−1^
DOPAC	2.17	4.53 10^−10^	9.74	2.09 10^−9^
5-HIAA	1.99	4.73 10^−10^	5.33	1.27 10^−9^
HVA	0.39	8.56 10^−11^	4.79	1.05 10^−9^
NE	0.16	3.81 10^−11^	3.56	8.42 10^−10^
E	0.43	9.37 10^−11^	3.63	7.93 10^−10^
L-DOPA	0.13	2.71 10^−11^	2.58	5.23 10^−10^
MT	5.68	9.78 10^−10^	6.11	1.05 10^−9^
DA	0.95	2.49 10^−10^	1.93	5.04 10^−10^
5-HT	0.42	9.57 10^−11^	0.57	1.30 10^−10^

The detection and quantification limits are close to the nanomolar range, allowing for very fine quantification of biological samples, particularly from artificial cerebrospinal fluid continuously perfused through the intracerebral microdialysis probes. Compared to the bibliography, these previous values were lower or close to most of HPLC-ECD studies [[27], [28], [29], [30]]. In this sense, it would be possible to reduce the sampling time in microdialysis to achieve better resolution. However, it should be noted that the use of a mass spectrometry detector remains a major advantage in compound quantification [[31], [32], [33]].

## 4 Conclusion

The combination of a Doehlert experimental design with chemometric analyses such as PLS-DA and Pearson correlation enables understanding the chromatographic behavior of a compound mixture. In our study, the optimization of elution conditions was achieved with the assistance of a new combination of chemometric tools allowing simultaneous enhancement of multiple parameters to optimize compound elution: short elution times, maximization of column efficiency, and reduction of peak asymmetry. This optimization combination results in lower compound quantification limits.

The example presented in this article on the improvement of neurotransmitter and metabolite detection and quantification was successfully carried out. It would be possible to further increase detection efficiency by using reverse-phase columns with smaller pore diameters to sharpen the peaks. The use of a mass spectrometer could also enhance the quantification of compounds.

This strategy of implementing an elution optimization protocol can be extended to other areas of chemical analysis, allowing for an understanding of the influence of chromatographic parameters on the physicochemical behavior of the studied compounds.

## CRediT authorship contribution statement

**Camille Rosier:** Writing – review & editing, Investigation, Formal analysis. **Bruno P. Guiard:** Writing – review & editing, Supervision, Funding acquisition. **Guillaume Gotti:** Writing – original draft, Supervision, Methodology, Investigation, Formal analysis.

## Declaration of competing interest

The authors declare that they have no known competing financial interests or personal relationships that could have appeared to influence the work reported in this article.
