# Supplementary material for: Application of Doehlert design combined with chemometrics tools: Example of the optimization of the elution of neurotransmitters and metabolites by HPLC
Source: Heliyon. 2025 Feb 14;11(4):e42690. doi: 10.1016/j.heliyon.2025.e42690 (PMC11883356; doi:10.1016/j.heliyon.2025.e42690)
Supplement: Multimedia component 1 [file mmc1.docx]

Supporting information file 1. Doehlert coefficients (X values).

|  | ACN  (%) | MeOH  (%) | pH | SDS  (mol L^-1^) | Citric acid  (mol L^-1^) |
| --- | --- | --- | --- | --- | --- |
| 1 | 0 | 0 | 0 | 0 | 0 |
| 2 | 1 | 0 | 0 | 0 | 0 |
| 3 | 0.5 | 0.866 | 0 | 0 | 0 |
| 4 | -0.5 | 0.866 | 0 | 0 | 0 |
| 5 | -1 | 0 | 0 | 0 | 0 |
| 6 | -0.5 | -0.866 | 0 | 0 | 0 |
| 7 | 0.5 | -0.866 | 0 | 0 | 0 |
| 8 | 0.5 | 0.289 | 0.816 | 0 | 0 |
| 9 | -0.5 | 0.289 | 0.816 | 0 | 0 |
| 10 | 0 | -0.577 | 0.816 | 0 | 0 |
| 11 | 0.5 | -0.289 | -0.816 | 0 | 0 |
| 12 | -0.5 | -0.289 | -0.816 | 0 | 0 |
| 13 | 0 | 0.577 | -0.816 | 0 | 0 |
| 14 | 0.5 | 0.289 | 0.204 | 0.791 | 0 |
| 15 | -0.5 | 0.289 | 0.204 | 0.791 | 0 |
| 16 | 0 | -0.577 | 0.204 | 0.791 | 0 |
| 17 | 0 | 0 | -0.612 | 0.791 | 0 |
| 18 | 0.5 | -0.289 | -0.204 | -0.791 | 0 |
| 19 | -0.5 | -0.289 | -0.204 | -0.791 | 0 |
| 20 | 0 | 0.577 | -0.204 | -0.791 | 0 |
| 21 | 0 | 0 | 0.612 | -0.791 | 0 |
| 22 | 0.5 | 0.289 | 0.204 | 0.158 | 0.775 |
| 23 | -0.5 | 0.289 | 0.204 | 0.158 | 0.775 |
| 24 | 0 | -0.577 | 0.204 | 0.158 | 0.775 |
| 25 | 0 | 0 | -0.612 | 0.158 | 0.775 |
| 26 | 0 | 0 | 0 | -0.632 | 0.775 |
| 27 | 0.5 | -0.289 | -0.204 | -0.158 | -0.775 |
| 28 | -0.5 | -0.289 | -0.204 | -0.158 | -0.775 |
| 29 | 0 | 0.577 | -0.204 | -0.158 | -0.775 |
| 30 | 0 | 0 | 0.612 | -0.158 | -0.775 |
| 31 | 0 | 0 | 0 | 0.632 | -0.775 |
