# Supplementary material for: Application of Doehlert design combined with chemometrics tools: Example of the optimization of the elution of neurotransmitters and metabolites by HPLC
Source: Heliyon. 2025 Feb 14;11(4):e42690. doi: 10.1016/j.heliyon.2025.e42690 (PMC11883356; doi:10.1016/j.heliyon.2025.e42690)
Supplement: Multimedia component 2 [file mmc2.docx]

Supporting information file 2. Table of standards concentrations for determination of LOD and LOQ.

A first mother solution comprising a mixture of the compounds of interest is prepared. The final concentration is 1 10^-4^ g L^-1^. A solution of ascorbic acid is added (1 mg mL^-1^) to prevent oxidation of the compounds. Its final concentration of compounds is 50 µg mL^-1^. This solution has been called S_1_. A daughter solution is prepared from solution S_1_. The final concentration of compounds is 1.33 10^-5^ g L^-1^. This solution is called S_2_. Finally, the different levels of concentration have been prepared from both solutions as reported in the following table.

| Level | $V_{S_{1}}(\mu L)$ | $V_{S_{2}}(\mu L)$ | Final V (µL)* | Final concentration  (g L^-1^)** | Final concentration  (pg / 25µL)** |
| --- | --- | --- | --- | --- | --- |
| 1 | - | 0 | 1502 | 0 | 0.000 |
| 2 | - | 2 | 1504 | 1.77 10^-8^ | 0.443 |
| 3 | - | 4 | 1506 | 3.54 10^-8^ | 0.885 |
| 4 | - | 6 | 1508 | 5.31 10^-8^ | 1.326 |
| 5 | - | 8 | 1510 | 7.06 10^-8^ | 1.766 |
| 6 | - | 10 | 1512 | 8.82 10^-8^ | 2.205 |
| 7 | - | 12 | 1514 | 1.06 10^-7^ | 2.642 |
| 8 | - | 14 | 1516 | 1.23 10^-7^ | 3.078 |
| 9 | - | 16 | 1518 | 1.41 10^-7^ | 3.513 |
| 10 | - | 18 | 1520 | 1.58 10^-7^ | 3.947 |
| 11 | - | 20 | 1522 | 1.75 10^-7^ | 4.380 |
| 12 | - | 25 | 1527 | 2.18 10^-7^ | 5.457 |
| 13 | - | 30 | 1532 | 2.61 10^-7^ | 6.527 |
| 14 | - | 40 | 1542 | 3.46 10^-7^ | 8.647 |
| 15 | - | 50 | 1552 | 4.30 10^-7^ | 10.739 |
| 16 | - | 60 | 1562 | 5.12 10^-7^ | 12.804 |
| 17 | - | 70 | 1572 | 5.94 10^-7^ | 14.843 |
| 18 | - | 80 | 1582 | 6.74 10^-7^ | 16.856 |
| 19 | - | 90 | 1592 | 7.54 10^-7^ | 18.844 |
| 20 | - | 100 | 1602 | 8.32 10^-7^ | 20.807 |
| 21 | - | 110 | 1612 | 9.10 10^-7^ | 22.746 |
| 22 | - | 120 | 1622 | 9.86 10^-7^ | 24.661 |
| 23 | - | 130 | 1632 | 1.06 10^-6^ | 26.552 |
| 24 | - | 140 | 1642 | 1.14 10^-6^ | 28.421 |
| 25 | - | 150 | 1652 | 1.21 10^-6^ | 30.266 |
| 26 | - | 160 | 1662 | 1.28 10^-6^ | 32.090 |
| 27 | - | 170 | 1672 | 1.36 10^-6^ | 33.892 |
| 28 | - | 180 | 1682 | 1.43 10^-6^ | 35.672 |
| 29 | - | 190 | 1692 | 1.50 10^-6^ | 37.431 |
| 30 | - | 200 | 1702 | 1.57 10^-6^ | 39.170 |
| 31 | - | 250 | 1752 | 1.90 10^-6^ | 47.565 |
| 32 | - | 300 | 1802 | 2.22 10^-6^ | 55.494 |
| 33 | - | 350 | 1852 | 2.52 10^-6^ | 62.995 |
| 34 | 40 | - | 1542 | 2.59 10^-6^ | 64.851 |
| 35 | 50 | - | 1552 | 3.22 10^-6^ | 80.541 |
| 36 | 60 | - | 1562 | 3.84 10^-6^ | 96.031 |
| 37 | 70 | - | 1572 | 4.45 10^-6^ | 111.323 |
| 38 | 80 | - | 1582 | 5.06 10^-6^ | 126.422 |
| 39 | 90 | - | 1592 | 5.65 10^-6^ | 141.332 |
| 40 | 100 | - | 1602 | 6.24 10^-6^ | 156.055 |
| 41 | 110 | - | 1612 | 6.82 10^-6^ | 170.596 |
| 42 | 120 | - | 1622 | 7.40 10^-6^ | 184.957 |
| 43 | 130 | - | 1632 | 7.97 10^-6^ | 199.142 |
| 44 | 140 | - | 1642 | 8.53 10^-6^ | 213.155 |
| 45 | 150 | - | 1652 | 9.08 10^-6^ | 226.998 |
| 46 | 160 | - | 1662 | 9.63 10^-6^ | 240.674 |
| 47 | 170 | - | 1672 | 1.02 10^-5^ | 254.187 |
| 48 | 180 | - | 1682 | 1.07 10^-5^ | 267.539 |
| 49 | 190 | - | 1692 | 1.12 10^-5^ | 280.733 |
| 50 | 200 | - | 1702 | 1.18 10^-5^ | 293.772 |
| 51 | 250 | - | 1752 | 1.43 10^-5^ | 356.735 |
| 52 | 300 | - | 1802 | 1.66 10^-5^ | 416.204 |
| 53 | 350 | - | 1852 | 1.89 10^-5^ | 472.462 |
| 54 | 400 | - | 1902 | 2.10 10^-5^ | 525.762 |

* The final volume includes the addition of a supplementary 2 µL of the antioxidant solution of ascorbic acid at 1 mg mL^-1^ and 1500 µL of ultra-pure water.

** The different concentrations reported in the table will be experimentally adjusted according to the weighed quantities. The concentrations presented are indicative.
