# Supplementary material for: Application of Doehlert design combined with chemometrics tools: Example of the optimization of the elution of neurotransmitters and metabolites by HPLC
Source: Heliyon. 2025 Feb 14;11(4):e42690. doi: 10.1016/j.heliyon.2025.e42690 (PMC11883356; doi:10.1016/j.heliyon.2025.e42690)
Supplement: Multimedia component 3 [file mmc3.docx]

Supporting information file 3. Chromatograms and chromatographic data of interested compounds obtained with several ratio of ACN/MeOH/H_2_O with pH = 1.65.

- ACN/H_2_O (15/85; v/v). Elution duration between (A) 0 and 20 minutes and (B) 0 and 2 minutes.

E & L-DOPA

MT

5-HIAA

HVA

DOPAC

- ACN/MeOH/H_2_O (11.25/3.25/85.5; v/v/v). Elution duration between (A) 0 and 25 minutes and (B) 0 and 2 minutes.

- ACN/MeOH/H_2_O (7.5/6.5/86; v/v/v). Elution duration between (A) 0 and 30 minutes and (B) 0 and 2 minutes.
- MeOH/H_2_O (13/87; v/v). Elution duration between (A) 0 and 40 minutes and (B) 0 and 2 minutes.
- Chromatographic data.

Retention time

| Compounds | ACN/H_2_O  (15/85; v/v) | ACN/MeOH/H_2_O  (11.25/3.25/85.5; v/v/v) | ACN/MeOH/H_2_O  (7.5/6.5/86; v/v/v) | MeOH/H_2_O  (13/87; v/v) |
| --- | --- | --- | --- | --- |
| DOPAC | 0.529 | 0.525 | 0.522 | 0.509 |
| 5-HIAA | 0.588 | 0.592 | 0.595 | 0.611 |
| HVA | 0.668 | 0.684 | 0.687 | 0.727 |
| NE | 2.782 | 3.194 | 3.464 | 3.878 |
| E | 3.763* | 4.356 | 4.847 | 5.402 |
| L-DOPA | 3.763* | 4.784 | 5.630 | 7.460 |
| MT | 4.450 | 6.050 | 7.745 | 11.868 |
| DA | 6.668 | 8.477 | 10.371 | 13.673 |
| 5-HT | 14.815 | 20.528 | 24.874 | 35.127 |

* Coelution of compounds

Number of theorical plates (N)

| Compounds | ACN/H_2_O  (15/85; v/v) | ACN/MeOH/H_2_O  (11.25/3.25/85.5; v/v/v) | ACN/MeOH/H_2_O  (7.5/6.5/86; v/v/v) | MeOH/H_2_O  (13/87; v/v) |
| --- | --- | --- | --- | --- |
| DOPAC | 5539 | 5345 | 4797 | 3279 |
| 5-HIAA | 4337 | 3812 | 3558 | 2540 |
| HVA | 4992 | 4613 | 4278 | 3381 |
| NE | 8482 | 8493 | 8594 | 8381 |
| E | 8616 | 9290 | 9117 | 9130 |
| L-DOPA | 8597 | 9259 | 8796 | 9306 |
| MT | 8958 | 9627 | 9393 | 8219 |
| DA | 12398 | 9735 | 9748 | 9306 |
| 5-HT | 10124 | 9777 | 10003 | 10059 |
| **Mean** | **8005** | **7772** | **7587** | **7136** |

Peak asymmetry

| Compounds | ACN/H_2_O  (15/85; v/v) | ACN/MeOH/H_2_O  (11.25/3.25/85.5; v/v/v) | ACN/MeOH/H_2_O  (7.5/6.5/86; v/v/v) | MeOH/H_2_O  (13/87; v/v) |
| --- | --- | --- | --- | --- |
| DOPAC | 2.02 | 1.51 | 1.62 | 1.83 |
| 5-HIAA | 1.51 | 1.67 | 1.61 | 1.76 |
| HVA | 1.41 | 1.45 | 1.41 | 1.50 |
| NE | 1.14 | 1.11 | 1.09 | 1.11 |
| E | 1.09 | 1.05 | 1.03 | 1.09 |
| L-DOPA | 1.08 | 1.07 | 1.02 | 1.05 |
| MT | 1.02 | 1.04 | 1.07 | 1.05 |
| DA | 1.05 | 1.05 | 1.10 | 1.07 |
| 5-HT | 1.05 | 1.11 | 1.08 | 1.13 |
| **Mean** | **1.26** | **1.23** | **1.22** | **1.29** |

Resolution. The presented value is between the compound of the line and the next one.

| Compounds | ACN/H_2_O  (15/85; v/v) | ACN/MeOH/H_2_O  (11.25/3.25/85.5; v/v/v) | ACN/MeOH/H_2_O  (7.5/6.5/86; v/v/v) | MeOH/H_2_O  (13/87; v/v) |
| --- | --- | --- | --- | --- |
| DOPAC | 1.89 | 1.92 | 1.99 | 2.29 |
| 5-HIAA | 2.06 | 2.15 | 2.40 | 2.34 |
| HVA | 26.84 | 28.23 | 28.98 | 28.89 |
| NE | 6.85 | 7.66 | 7.83 | 7.85 |
| E | Coelution with L-DOPA | 1.70 | 3.52 | 7.51 |
| L-DOPA | 4.04 | 6.12 | 7.70 | 10.62 |
| MT | 10.40 | 8.52 | 7.01 | 3.45 |
| DA | 19.67 | 19.62 | 20.39 | 21.89 |
| 5-HT |  |  |  |  |
