# Supplementary material for: Application of Doehlert design combined with chemometrics tools: Example of the optimization of the elution of neurotransmitters and metabolites by HPLC
Source: Heliyon. 2025 Feb 14;11(4):e42690. doi: 10.1016/j.heliyon.2025.e42690 (PMC11883356; doi:10.1016/j.heliyon.2025.e42690)
Supplement: Multimedia component 4 [file mmc4.docx]

Supporting information file 4. Calibration curves of (A) DOPAC, (B) 5-HIAA, (C) HVA, (D) NE, (E) E, (F) L-DOPA, (G) MT, (H) DA and (I) 5-HT.

| **A** |  |
| --- | --- |
|  |  |
|  |  |
|  |  |
|  |  |
